# The Treatment of Tabes Dorsalis
1Dr. Maurice Faure, Lancet, June 18, 1904.


**Published:** 1904-07-09

**Authors:** 


### THE TREATMENT OF TABES DORSALIS.[1]

THE physician must in every case take into consideration such causes as syphilis, overwork, alcoholism, tobacco poisoning, influenza, tuberculosis, and the like. In view of the predominant part played by syphilis, anti-syphilitic treatment ought always to be tried, at all events in the early stage; but it ought not to be maintained as a routine or continued in patients with marked tabetic lesions and with impaired general health. By such a course an additional poison is introduced into the organism and the nervous disorders are aggravated. In estimating the effects of any scheme of treatment and also in framing a prognosis it must be remembered that the disease is not necessarily and invariably progressive. In about a third of the cases there is a steady advance of the disease, in another fourth there is arrest or improvement, whilst in the remainder the movement is slow with occasional inconveniences and prolonged periods of quiescence. The tabetic must, it is true, often modify his activities and live the life of an invalid rather than that of a vigorous man. But he may be guarded against the dangers of complications and there is even hope that he may regain a considerable measure of good health. It is the first duty of the physician to disabuse the patient's mind of any expectation of a rapid cure, but equally he must be preserved from despondency and despair. The functions of the bladder and intestine must be carefully watched, ample nourishment and sleep must be secured, and physical and mental overwork be avoided. Every year one or more months should be spent at a sanatorium where special exercises to train the respiratory system and the locomotive powers should be practised. Thermal hydrotherapeutic measures may be associated with these. But success is only to be expected by those who will continue this plan of life for years. If adequate treatment is adopted and sustained the great majority of tabetics will at least find their lot a much more bearable one than is commonly believed. Regarding the so-called "intensive" treatment of tabes dorsalis and general paralysis attention may be given to the experience of Lemoine of Lille,[2] who advocates the hypodermic injection of an organic salt of mercury in large doses, continued over prolonged periods. He has published a number of cases in which benzoate of mercury continued in daily doses of two to three centigrammes over several months has been followed by marked improvement, if not by complete cure. Leredde,[3] of Paris, also supports this recommendation and reports a considerable experience in its favour. Even in general paralysis, a disease commonly regarded as practically hopeless, he can show some very striking successes. He urges that both in tabes dorsalis and in general paralysis the treatment should commence at the earliest possible date; that for vigorous adults from one-half to three-quarters of a grain of benzoate of mercury in normal saline solution should be given daily by hypodermic injection; and that signs of intolerance, such as indigestion, loss of flesh, and febrile reaction, indicate overdosage. The dose in any case should only be slowly increased, and throughout the treatment attention should be paid to the state of the mouth and gums.

[1] Dr. Maurice Faure, Lancet, June 18, 1904. [2] Revue Neurologique, July 30, 1902 (Lancet, Sept. 6, 1902). [3] Philadelphia Med. Journ., April 18, 1903 (Lancet, June 6, 1903).

